# Effects of Integrating Family Planning With Maternal, Newborn, and Child Health Services on Uptake of Voluntary Modern Contraceptive Methods in Rural Pakistan: Protocol for a Quasi-experimental Study

**DOI:** 10.2196/35291

**Published:** 2022-03-08

**Authors:** Zahid Ali Memon, Sophie Reale, Wardah Ahmed, Rachael Spencer, Talib Hussain Lashari, Zulfiqar Bhutta, Hora Soltani

**Affiliations:** 1 Centre of Excellence in Women and Child Health Aga Khan University Karachi Pakistan; 2 Health Research Institute Sheffield Hallam University Sheffield United Kingdom; 3 College of Health, Wellbeing and Life Sciences Sheffield Hallam University Sheffield United Kingdom; 4 Department of Nursing and Midwifery Sheffield Hallam University Sheffield United Kingdom; 5 Population Welfare Department Karachi Pakistan

**Keywords:** family planning, integrated health services, contraceptive prevalence rate, modern contraceptive prevalence rate, modern contraceptive method, rural Pakistan

## Abstract

**Background:**

The uptake of modern contraceptive methods (MCMs) remains low, with 25% of women reporting their use in Pakistan. The overarching interventions covering service delivery platforms at facility and community levels necessitate the integration of family planning (FP) with maternal, newborn, and child health (MNCH) services.

**Objective:**

The main aim of this study is to evaluate the impact of an integrated FP-MNCH service delivery model to increase coverage of MCMs in rural Pakistan. Moreover, we aim to measure the level of effectiveness of interventions regarding the uptake of MCMs.

**Methods:**

A quasi-experimental, sequential, mixed methods study design with pre- and postevaluation will be adopted to evaluate the impact of integration of FP with MNCH services. The interventions include the following: (1) capacity strengthening of health care providers, including technical trainings; training in counseling of women who attend immunization centers, antenatal care (ANC) clinics, and postnatal care (PNC) clinics; and provision of job aids; (2) counseling of women and girls attending ANC, PNC, and pediatric clinics; (3) ensuring sustained provision of supplies and commodities; (4) community engagement, including establishing adolescent-friendly spaces; and (5) use of District Health Information System data in decision-making. Descriptive statistics will be used to estimate prevalence (ie, proportions) and frequencies of outcome indicators. A univariate difference-in-difference analytical approach will be used to estimate the effect of the interventions. In addition, a Blinder-Oaxaca decomposition analysis will be conducted to identify and quantify determinants of the modern contraceptive prevalence rate.

**Results:**

The intervention phase began in July 2021 and will run until June 2022. The impact assessment will be conducted from July to September 2022.

**Conclusions:**

This project will evaluate the impact of integrating FP with MNCH services. Furthermore, this study will identify the drivers and barriers in uptake of MCMs and will simultaneously help in modifying the interventional strategies that can be scaled up through existing service delivery platforms within the public and private sectors, according to the local sociocultural and health system context.

**Trial Registration:**

ClinicalTrials.gov NCT05045599; https://clinicaltrials.gov/ct2/show/NCT05045599

**International Registered Report Identifier (IRRID):**

DERR1-10.2196/35291

## Introduction

### Background

The scaling up of family planning (FP) programs has yielded a multitude of benefits in terms of improving health outcomes and paving opportunities for poverty reduction and women’s empowerment. FP programs have effectively contributed to a significant reduction of 32% of all maternal deaths and 10% of all child deaths. Moreover, these programs enhance women’s empowerment and gender equity, and, within that, they are aimed at achieving a higher level of universal female primary education. Over the past four decades, FP programs have led to a globally increased contraceptive prevalence rate (CPR), from 10% to 60%, and a substantial reduction in fertility rate from 6 to 3 children per woman in low- and middle-income countries [1,2].

Modern contraceptive methods (MCMs), such as condoms, contraceptive pills, injectables, and intrauterine devices, serve as significant measures of fertility control [1]. There is a strong body of evidence suggesting that providing a woman with opportunities to opt for the MCM of her choice through a continuum of care is viable and cost-effective, not only at the individual level but also at the broader health system level [2,3].

In Pakistan, FP programs started in the mid-1960s [1]. Later, a Lady Health Worker (LHW) outreach program was initiated in 1994, which primarily focused on FP services at the national level. Pakistan has been a signatory on various international commitments to improve access to reproductive health services, including the following: (1) the Millennium Development Goals (2000), aimed at increasing the CPR from 12% in 1990 to 55% in 2015 [4]; (2) the London Summit on Family Planning 2012, aimed at raising the CPR to 55% by 2020; and (3) the Sustainable Development Goals (2015), aimed at achieving universal access to reproductive health services by 2030. Pakistan has made considerable progress over time, yet these targets have not been fully reached [5].

Generally, the provincial government’s Population Welfare Department and the Department of Health are both responsible for translating the government’s vision of health and FP. However, the Department of Health has lagged behind in taking ownership of the FP agenda to fully optimize the resources to achieve the objectives [6]. The government’s FP2020 agenda invigorated efforts to accelerate progress in achieving higher use of MCMs. Political commitment at the national level and at the Sindh province level culminated in appointing the same minister to the Department of Health and the Population Welfare Department to ensure functional integration in both departments. Moreover, the FP2030 Secretariat has been created to bring all stakeholders onto a single platform and provide facilitation for nongovernmental organizations (NGOs) and private sector entities that are working in the FP arena.

Several collaborative initiatives have contributed to increased MCM uptake in Pakistan, as demonstrated by a nearly three-fold increase in the CPR from 13% to 34% between 1990 and 2018 (0.5% annual increment) [7-9]. However, there still remains a large unmet need for FP, toward which progress has been slow and in some cases stagnant.

A review of the existing data report low use of MCMs at 25% among women aged 15 to 49 years and 7% among adolescents aged 15 to 19 years in Pakistan [7]. This indicates a high unmet need of FP, specifically among currently married women (17%). For example, out of all pregnancies in Pakistan, 46% are unintended and unsafe, and the abortion rate remains high at 50 per 1000 women [7]. It is noteworthy that 78% of nonusers of MCMs are women aged 15 to 49 years who have never had discussions regarding FP methods with any health care provider at the community or facility levels [7]. Further, of those who give birth, many do not receive (1) standard antenatal care (ANC), postnatal care (PNC), or quality services at the time of delivery or (2) appropriate management for mother and newborn complications [10].

This is the result of various barriers that have evolved within the broader political, social, and cultural context of Pakistan. A deeper understanding of these barriers and determinants is required to identify gaps within the health system and expand the coverage of FP and maternal, newborn, and child health (MNCH) services [1,11]. From the demand side, literature suggests that women’s empowerment, gender equality, and religious obstacles are contributing factors that heavily influence the uptake of contraceptive methods [12]. However, a further exploration is required at the local level in terms of community perspectives of, resistance to, and attitudes toward FP and contraceptive uptake. Within the country, people living in the lower wealth quintiles (below 25%) and residing in rural areas have a lower literacy level, with young women experiencing a great unmet need for FP services and products [13]. This has translated into poor acknowledgement and adoption of practices relating to healthy timing and spacing of pregnancies and, consequently, higher fertility rates [14,15]. Preference for sons and a lack of husbands’ participation also count as prominent factors in the context of Pakistan that present key barriers to the uptake of FP methods [16,17]. Studies further report that the involvement of supportive male family members has effectively increased the rate of voluntary uptake of contraceptive methods [17]. Given that the bulk of Pakistan’s population is comprised of youth, the need for adolescent-focused interventions to evaluate and improve reproductive health outcomes at scale is urgently needed [8,18].

On the supply side, the literature reports lack of access to information; lack of access to health care service delivery points, especially in rural communities; and fear of side effects as key barriers to MCM uptake. For those who do adopt an MCM, method discontinuation due to poor quality of services provided, attitudes of health care providers, and cost-bearing during treatment of side effects [9,13,18,19] are key factors in low contraceptive prevalence. Considering FP as the major factor in improving health indicators throughout the course of women’s and children’s lives [20], thereby building trust in government services, making needs-based alignment of FP programs in the country would substantially affect the resistance to opting for contraception [21].

Given these barriers, designing and evaluating integrated FP programs that are based on needs and delivered through existing service delivery platforms have the potential to improve the health and well-being of women and children in Pakistan. The integration of FP with immunization in Malawi resulted in a 14% increase in the uptake of FP methods. Parents experienced greater feasibility marked by reductions in time lost and transport costs as well as access to greater awareness and knowledge while accessing services on the same day. Similarly, health care providers expressed that this integration enhanced their skills, knowledge, and competence [20]. Similar results were found in Liberia and Rwanda, with an emphasis on maintaining privacy for couples in public health settings. The integration of the immunization schedule, with extended postpartum FP, resulted in an increase in uptake [22,23]. In order for the successful and equity-based implementation of integrated services, it was reported that following standard protocols for provision of care and training of health care providers at facility and community levels was necessary [24].

Moreover, a recent study conducted during the COVID-19 pandemic in Ethiopia examined the integration of FP with maternal health care services, including ANC, delivery, PNC, and immunization. The results showed a 6% increase in the uptake of integrated FP services, which is significant in a low-resource setting, given the widespread effect of the global pandemic [25].

The literature has also shown that interventions focusing on community outreach programs and interpersonal communications increase social acceptance of FP methods [26]. However, home-based counseling alone is not sufficient for the uptake and continuation of FP methods [27]; developing linkages with health facilities, maintaining privacy at a health facility, and delivering services in a socioculturally sensitive manner is also important.

In line with this, efforts have been made that involve facility- and community-level health care providers for the provision of MNCH services as the primary mandate of the National MNCH Program. However, there are still deficiencies at the interfacility and intrafacility levels, for example, (1) a lack of coordination among departments, such as pediatrics and gynecology and obstetrics; (2) a lack of management-level coordination with frontline health workers; (3) a lack of equipment and logistics management manifested as an imbalance in demand and supply; and (4) a lack of overall governing bodies [6,28]. Thus, overarching interventions covering service delivery platforms at facility and community levels necessitate the integration and scaling up of FP and MNCH services.

### Implementation Context

**Figure 1:**
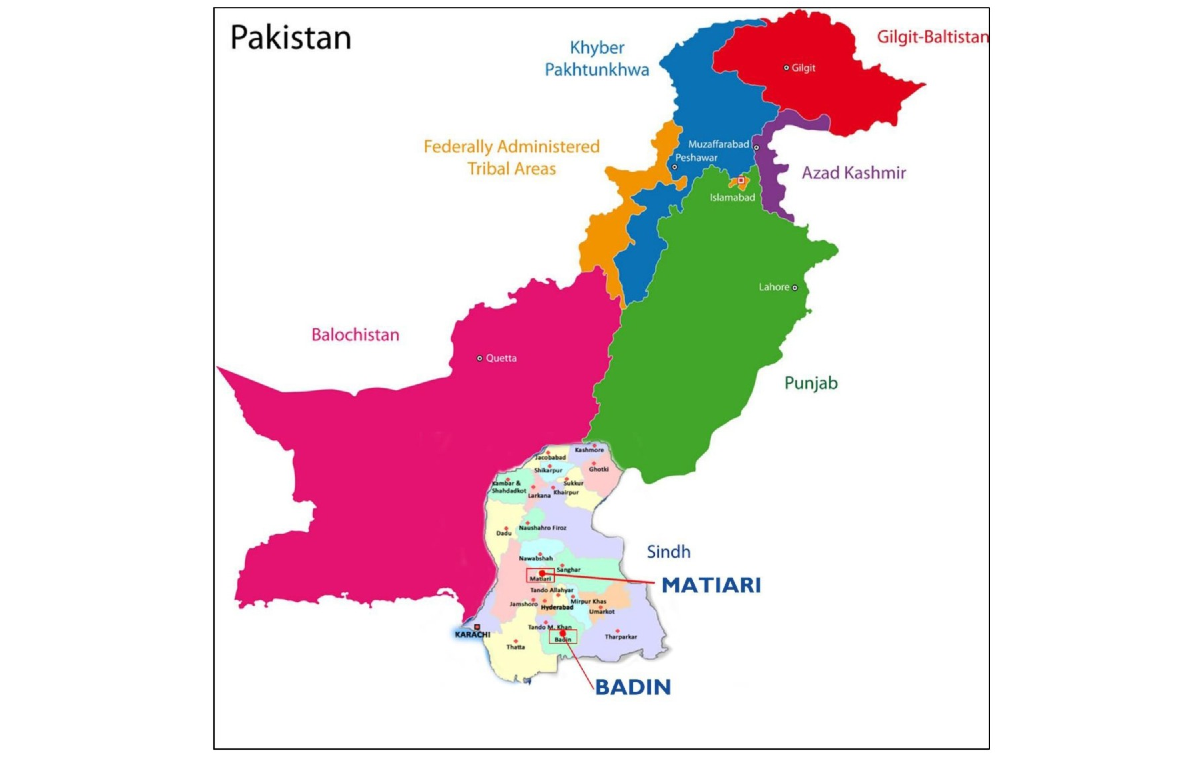
Map of Pakistan showing the districts of Badin (control) and Matiari (intervention) in Sindh province.

Pakistan is comprised of four provinces (ie, Punjab, Sindh, Khyber Pakhtunkhwa, and Balochistan), two autonomous territories (ie, Gilgit-Baltistan and Azad Jammu Kashmir), and a federal territory (ie, Islamabad); these collectively encompass 150 districts [29]. This study will be conducted in two districts of Sindh province (Figure 1). Sindh has the highest rural-urban difference in fertility rates: 4.7 and 2.9 per woman, respectively. Median age at first birth is 23 years, with only 18% of this age group receiving any contraceptive method. Overall, modern CPR (mCPR) in Sindh is lower (24% vs 26%) and unmet needs are higher (22% vs 17%) as compared to national-level statistics [30].

The public health system consists of a three-tiered health delivery system comprised of domiciliary and outreach services rolled out through LHWs and primary- and secondary-level health care facilities in each district [2]. Each LHW has a catchment population of approximately 1000 to 1500 people. They provide educational, preventive, and promotive services as well as some aspects of curative services in the community. They provide MNCH and FP services to eligible families.

### Theoretical Framework

**Figure 2:**
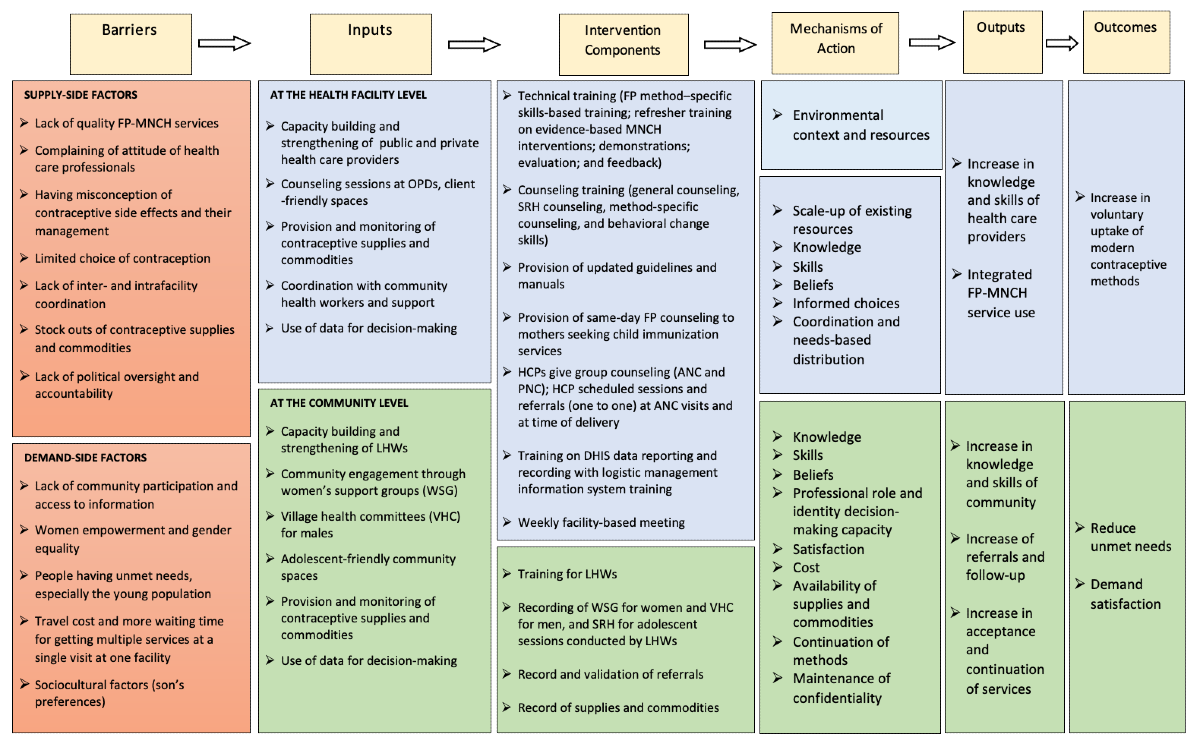
Theoretical Framework: integrated FP-MNCH services. ANC: antenatal care; DHIS: District Health Information System; FP: family planning; HCP: health care provider; LHW: Lady Health Worker; MNCH: maternal, newborn, and child health; OPD: outpatient department; PNC: postnatal care; SRH: sexual and reproductive health; VHC: village health committee; WSG: women support group.

The theoretical underpinning of behavior change will be based on the Theoretical Domains Framework (TDF), version 2.0. The TDF will be applied to provide an in-depth exploration and understanding of factors regarding the demand and supply side and their interaction with and influences on FP uptake [31,32]. This project aims to implement a complex intervention (Figure 2) within health facilities and their catchment communities. This complex intervention includes a series of strategies involving community engagement by extensive community mobilization, availability of trained staff, and sustainable supply of commodities with the required recording and reporting system. Continuous process monitoring and quality assurance will help to replicate the success and address possible barriers during implementation of the intervention. The mechanism of action built on the TDF adopts domains and constructs, including knowledge, skills, beliefs, and intentions. Furthermore, the TDF provides a detailed understanding of complex behavior; thus, it will be used to evaluate the impact of complex interventions and strategies.

### Research Question

The research question that will be answered by this study is as follows: What is the impact of integrating FP with MNCH services on the uptake of voluntary MCMs in a rural district of Sindh province, Pakistan?

### Aim and Objectives

The aim of this study will be to evaluate the impact of an integrated FP-MNCH service delivery model to increase coverage of MCMs in rural Pakistan.

The objectives of this study are as follows:

- To gain an understanding of the cultural and health service delivery contexts to inform a socioculturally appropriate and acceptable intervention package scalable in rural Pakistan
- To implement the intervention package at health facilities and outreach communities through existing public and private sector resources
- To measure the impact and level of effectiveness of interventions on the uptake MCMs
- To identify and quantify the drivers of improved uptake of voluntary methods of FP, especially MCMs.

## Methods

### Study Design

A quasi-experimental, sequential, mixed methods study design with pre- and postevaluation is proposed to assess the impact of implementing an integrated delivery model on the uptake of MCMs in a rural district of Sindh province, Pakistan.

### Ethics Approval

Ethical clearance for this project was sought from the Ethical Review Committee of the Aga Khan University on June 26, 2020. The study protocol was approved by the National Bioethics Committee, Pakistan (ERC number 2021-3606-19065). The baseline assessment was completed in January 2021. The inception period included hiring and deployment of study staff and their training in the standard operating procedures of the project.

### Qualitative Component

#### Overview

The overall objective of the qualitative component is to understand the barriers that married women and girls who are at risk of an unwanted pregnancy face in accessing and using voluntary FP. The information will be used to inform the integrated intervention design and delivery platform. The qualitative component will include focus group discussions (FGDs) with married women of reproductive age (MWRA; 18-49 years) and adolescent girls and boys (15-19 years) to understand behaviors and community-level hindrances to acceptance and use of FP methods. Due to the sensitivity of the topics, separate FGDs will be conducted with male and female participants. In-depth interviews will also be conducted with service providers and health facility managers to understand facility-level and supply-side barriers to uptake of modern FP methods and services (see Multimedia Appendix 1 for sample sizes of FGDs and in-depth interviews).

A purposive sampling technique will be used. We aim to conduct 10 in-depth interviews and two FGDs, with 5 to 6 participants in each group. However, this will depend on reaching the theoretical point of saturation. A thematic approach will be used to analyze the data. The FGD guide for data collection will be based on the TDF.

#### Analysis of the Qualitative Component

Analysis will be done using NVivo software (version 11; QSR International). The TDF will be the guiding framework for coding and thematic analysis.

### Systematic Review

A systematic review of the existing research will be conducted to identify effective FP interventions and strategies that led to an increase in the uptake of MCMs. The research question will look at the impact of effective interventions and strategies on improving the uptake of MCMs in South Asian countries. Various databases (PubMed, Cochrane Database of Systematic Reviews, EBSCO CINAHL, Web of Science, ProQuest Dissertations & Theses, etc) will be used to collect articles published from January 2000 to June 2021. Covidence, a systematic review management program, will be employed to facilitate collaboration across the team. A meta-analysis will be used to synthesize the data from relevant studies into a single quantitative pooled estimate. The pooled estimate will be the outcome of the meta-analysis and will be explained by a forest plot. The meta-analysis will be done using RevMan (Review Manager) software (version 5.4.1; The Cochrane Collaboration). For categorical variables, odds ratios with 95% CIs will be reported; for continuous outcomes, mean differences or standard mean differences with 95% CIs will be reported.

### Quantitative Component

#### Overview

A nonrandomized, quasi-experimental study design with intervention and control arms is proposed to assess the impact of implementing a complex delivery model of interventions on the voluntary uptake of MCMs in two rural districts of Sindh. Two sequential face-to-face household surveys will be conducted before and after the 12-month implementation of the interventions [33]. The surveys will document the changes over time in mCPR alongside other outcomes of interest, such as unmet needs and demands satisfied.

#### Selection of Control District

We used the propensity score matching technique to select the control district (Table 1). This procedure estimates the probability and propensity that a study unit that has not received the intervention is similar at baseline to another unit from the intervention group, based on a set of key characteristics. As such, it reduces the problem of comparison across large numbers of key variables [34]. In the context of our study, this matching was done to select Badin as the control district to compare with the intervention district of Matiari (Figure 1). The variables used in generating propensity score matching included CPR, mCPR, unmet needs, ANC by skilled providers, deliveries by skilled birth attendants, the Human Development Index, and percentage of full immunization coverage. The district-level data of these indicators were taken from the Sindh Multiple Indicator Cluster Survey 2018 for all 23 rural districts (Multimedia Appendix 2).

**Table 1:** Selection of control district using propensity score matching.

District	Unmet needs, %	CPR^a^, %	Modern CPR, %	Fully immunized, %	Deliveries by skilled birth attendants, %	Antenatal care, %	Propensity score
Badin (control district)	17.9	28.1	28.1	45.6	61.9	82.0	0.36
Matiari (intervention district)	25.8	32.4	30.6	68.0	65.5	85.7	0.31

^a^CPR: contraceptive prevalence rate.

#### Sample Size Estimation

With 95% CI and 80% power, a sample size of 880 MWRA, 15 to 49 years, per arm is required to estimate an increase from 28.9% to 36.9% (ie, 8% increase) in the proportion of MCM uptake. The assumed design effect of 1.5 and a 7% nonresponse rate is accounted for in the sample size calculations [35].

#### Sampling and Recruitment Strategy

A two-stage random-sampling strategy will be employed to select the clusters and households with respondents (ie, MWRA, 15-49 years). A higher response rate has been projected, as the survey will be conducted at the field level on a face-to-face basis. The face-to-face method has been accredited in the literature as an effective way of eliciting a higher rate of response from target audiences [33]. Sampling will consider each cluster level as a stratum; each cluster will be comprised of 100 to 150 households in the catchment area of the health facility. Each cluster will be a primary sampling unit. At the first stage, clusters will be randomly selected from the sampling frame of each district. Each household will be a secondary sampling unit. At the second stage, 20 eligible households will be selected using systematic random sampling to reach the calculated sample size (ie, 880 MWRA, 15-49 years, per arm).

#### Outcomes

The primary outcome is the mCPR. The operational definition of the primary outcome can be found in Multimedia Appendix 3.

The secondary outcomes are as follows (see Multimedia Appendix 3 for their operational definitions):

- Unmet needs
- Demands satisfied
- Proportion of women showing positive attitudes toward FP visits where clients received integrated FP-MNCH services
- Proportion of referral completers who accepted an FP method
- Proportion of follow-up visits where clients received integrated FP-MNCH services
- Percentage of women who were satisfied with the services provided
- Percentage of staff scoring 70% or above on a knowledge assessment test using validated tools, at the facility and community levels
- Number of unwanted pregnancies and births
- Proportion of married women who used ANC and PNC services
- Proportion of institutional deliveries by skilled birth attendants.

#### Statistical Analysis of the Quantitative Component

The data will be collected using an Android app and will be uploaded in real time to the Aga Khan University server. Data cleaning will be performed by identifying errors and missing entries under the supervision of the senior data manager. Descriptive statistics will be used to estimate prevalence (ie, proportions) and frequencies of outcome indicators. A univariate difference-in-difference analytical approach will be used to estimate the effect of the interventions. In addition, a Blinder-Oaxaca decomposition analysis will be conducted to identify and quantify determinants of mCPR. Analyses will be done using Stata Statistical Software (version 16; StataCorp LLC). Weighted analysis will be performed by target age group for each cluster.

### Intervention Sites

The intervention will be implemented within existing health systems, including public and private health facilities, and among community-level health workers. The intervention duration will range from 12 to 18 months. Six public health facilities working under the Department of Health, 10 private health facilities, and all LHWs working in the catchment areas and along the referral pathway will be included in the Matiari district in Sindh (Table 2). A similar number of health facilities and outreach LHWs will be selected as control facilities and LHWs from the Badin district.

**Table 2:** Health system characteristics of the study districts.

District	Type of health facility, n				Lady Health Workers, n		Target population, n
	Secondary	Primary	Private				
Matiari (intervention district)	3	3	10	144		269,304	
Badin (control district)	3	3	10	170		604,888	

### Key Strategies to Implement Integrated FP-MNCH Interventions

An integrated FP-MNCH service delivery model will be implemented. FP will be integrated into the MNCH service delivery platforms of the intervention district’s public sector health care facilities, as follows:

1. ANC clinics: counseling on first ANC visit, counseling on follow-up ANC, and recruitment for postpartum FP uptake.
2. Labor and delivery rooms: counseling during admission to the health care facility. The focus of the counseling sessions will be on provision of information about the benefits of spacing of pregnancies for the mother and newborn, and information on different MCMs available and when to use them.
3. Pediatric outpatient departments: counseling of women of reproductive age and caregivers on the importance of FP and referral to the FP clinics.

In addition, LHWs will be encouraged to integrate FP into their routine MNCH activities at the household and community levels.

The proposed interventions and strategies based on the existing evidence are listed below; the strategies will be refined and finalized based on the findings of the qualitative research:

1. Capacity strengthening of health care providers serving at government secondary- and primary-level hospitals and LHWs serving in the facility catchments, as follows: (a) technical trainings; (b) training for counseling of women attending ANC, PNC, and pediatric clinics; and (c) provision of job aids.
2. Counseling of women and girls attending ANC, PNC, and pediatric clinics regarding the importance of FP and referral to the FP clinics.
3. Ensuring the sustained provision of supplies and commodities.
4. Community engagement, including adolescent and youth populations.
5. Use of District Health Information System data in decision-making.

## Results

The study was registered at ClinicalTrials.gov (NCT05045599). The baseline assessment was completed in January 2021. The inception period included hiring and deployment of study staff and their training in the standard operating procedures of the project. The intervention phase began in July 2021 and will run until June 2022. The impact assessment will be conducted from July to September 2022. Details of the study timeline are outlined in Multimedia Appendix 4.

## Discussion

Despite the knowledge and scalability of evidence-based interventions and commitment from the government and other NGOs to improve reproductive health, Pakistan still lags behind its regional counterparts in achieving accelerated progress in FP indicators. Addressing this lag requires exploring context-specific interventions and strategies to ensure affordable access to, and availability of, quality FP products, information, and services within existing service delivery platforms. This provides a prime opportunity to develop scalable and sustainable models that can reach and benefit the most vulnerable and marginalized populations across the country.

This project, through its qualitative components, seeks to deconstruct the sociocultural sensitivities and context of FP service delivery and uptake in Pakistan and further attempts to understand the barriers and facilitators to the voluntary uptake of FP services and improved health outcomes for women and young girls in this regard. Studies in India and Nigeria report a strong negative correlation between communities that harbor a conservative cultural and religious belief system and low demand and acceptance of contraceptive services and uptake [36]. These belief systems present a spectrum of factors, including women’s restrictions on movement, religious reservations regarding the use of contraceptives, and denial of women’s rights to decision-making and choices related to their health and well-being [36,37]. Many of these factors are present in the context of Pakistan and serve as barriers to FP access and uptake, specifically for women residing within communities in remote rural areas [21].

Therefore, identifying the local grassroots context and synchronizing it with FP intervention design and implementation strategies holds the key to addressing barriers to voluntary FP uptake faced by women in their day-to-day lives and achieving meaningful and effective coverage of FP interventions. Furthermore, studies conducted in Nepal and Bangladesh that reflect an FP demographic and landscape similar to that of Pakistan emphasize the efficiencies of adopting an integrated approach toward improving FP service delivery, quality, and uptake [30,38]. Strategically integrating FP services with services provided along the MNCH continuum of care has yielded significant impact in terms of increasing the prevalence of MCMs and their sustained use [39]. More specifically, the delivery of FP services to women at crucial points in the delivery of ANC, postpartum care, and PNC is highlighted in several studies in South and sub-Saharan Africa as a highly effective and feasible strategy to motivate new users of both short-term and long-term contraceptive methods [40,41].

Moreover, a plethora of quasi-experimental studies conducted within low- and middle-income countries, including Pakistan, concluded that FP intervention designs that cater to strengthening FP service delivery at both the facility and community levels are best equipped for success in the South Asian context [42-44]. Factors identified in the literature at the facility level include the following: capacity building of FP health care providers; strengthening supply chain and stock management of essential FP commodities and equipment; extending the scope of technical FP services and products, including surgical application of MCMs; and providing counseling services to women and family members at the facility level [45]. At the community level, the mobilization of community-based workers to generate awareness and demand for FP uptake is instituted through visiting households; educating and promoting FP among various community forums and groups; counseling women, men, couples, and families with regard to the importance and uptake of FP and MNCH; and establishing improved systems for referral and linkages [43,46]. This study, through its qualitative and quantitative components, will attempt to gauge both facility- and community-level factors that can be strengthened as part of the strategic implementation of the intervention to increase uptake of MCMs.

Therefore, this project provides a prime opportunity to generate evidence on effective interventions and strategies that are contextually relevant and sensitive. These strategies would improve access to FP information and services for the women of reproductive age and their family members who come into contact with the health system. Such care-seeking presents a premise of missed opportunities that can otherwise be effectively used by simply integrating FP information and services and making them available at the health facility level, in those facilities that provide MNCH services, and at the community level through LHWs and community worker mobilization.

Thus, the learnings from this study will provide a credible and robust foundation on which to design an effective intervention model that can eventually be scaled up to other districts, regions, and provinces across Pakistan. The lessons learned and the best practices emanating from this model will pave the way for evidence-informed FP program and policy making at the broader national level.

## Appendix

Multimedia Appendix 1 Sample sizes of focus group discussions and in-depth interviews.

Multimedia Appendix 2 Propensity score matching for selection of control district.

Multimedia Appendix 3 Operational definitions of the primary and secondary outcomes.

Multimedia Appendix 4 Project timeline.

## Abbreviations

ANC: antenatal care
CPR: contraceptive prevalence rate
FGD: focus group discussion
FP: family planning
LHW: Lady Health Worker
MCM: modern contraceptive method
mCPR: modern contraceptive prevalence rate
MNCH: maternal, newborn, and child health
MWRA: married women of reproductive age
NGO: nongovernmental organization
PNC: postnatal care
RevMan: Review Manager
TDF: Theoretical Domains Framework
