# Supplementary material for: Effects of Integrating Family Planning With Maternal, Newborn, and Child Health Services on Uptake of Voluntary Modern Contraceptive Methods in Rural Pakistan: Protocol for a Quasi-experimental Study
Source: JMIR Res Protoc. 2022 Mar 8;11(3):e35291. doi: 10.2196/35291 (PMC8941439; doi:10.2196/35291)
Supplement: Multimedia Appendix 1 [file resprot_v11i3e35291_app1.docx]

**Multimedia Appendix 1: Qualitative Sample Size**

Sample Size for Each Focus Group Discussion (FGD)

| **Districts** | **Participant Category** | | | | | **Total participants in each FGD** |
| --- | --- | --- | --- | --- | --- | --- |
|  | **Married Males** | **Married Females** | **Adolescent Boys** | **Adolescent Girls** | **Health care providers** |  |
|  |  |  |  |  |  |  |
|  |  |  |  |  |  |  |
| **Matiari** |  |  |  |  |  |  |
|  | 2 | 2 | 2 | 2 | 2 | 5-6 |

Sample Size In-depth Interviews (IDI)

| **Districts** | **Participant Category** | | | **Total IDIs** |
| --- | --- | --- | --- | --- |
|  | **Head of Department** | **Nurse/LHV^[[1]](#footnote-1)^/ CMW^[[2]](#footnote-2)^/ LHW** | **Gynecologist** |  |
|  |  |  |  |  |
|  |  |  |  |  |
| **Matiari** |  |  |  |  |
|  | 2 | 6 | 2 | 10 |

1. Lady Health Visitor [↑](#footnote-ref-1)
2. Community Midwife [↑](#footnote-ref-2)
