# Supplementary material for: Effects of Integrating Family Planning With Maternal, Newborn, and Child Health Services on Uptake of Voluntary Modern Contraceptive Methods in Rural Pakistan: Protocol for a Quasi-experimental Study
Source: JMIR Res Protoc. 2022 Mar 8;11(3):e35291. doi: 10.2196/35291 (PMC8941439; doi:10.2196/35291)
Supplement: Multimedia Appendix 2 [file resprot_v11i3e35291_app2.docx]

### **Multimedia Appendix 2: Selection of Control District using Propensity Score Matching (PSM) from 23 rural districts of Sindh**

| **SN** | **Districts** | **UMN** | **CPR** | **mCPR** | **HDI** | **FIMM** | **DSBA** | **ANC** | **Propensity Score(PSM)** |
| --- | --- | --- | --- | --- | --- | --- | --- | --- | --- |
| 1 | **Badin** | 17.9 | 28.1 | 28.1 | 41 | 45.6 | 61.9 | 82 | **0.36** |
| 2 | Sanghar | 21.7 | 24.9 | 24.9 | 49 | 59.4 | 59.4 | 76 | 0.19 |
| 3 | **Matiari** | 25.8 | 32.4 | 30.6 | 57 | 68 | 65.5 | 85.7 | **0.31** |
| 4 | Qamber Shahdadqot | 19.3 | 18.4 | 18.2 | 46 | 22.6 | 52.4 | 70.3 | 0.06 |
| 5 | Kashmore | 26.1 | 16 | 15.7 | 47 | 9.7 | 35.9 | 63.5 | 0.12 |
| 6 | Jacobabad | 22.8 | 19.9 | 16.9 | 44 | 18 | 51.9 | 59.5 | 0.00 |
| 7 | Larkana | 20.6 | 26.7 | 24.3 | 62 | 41 | 55.8 | 70.3 | 0.04 |
| 8 | Shikarpur | 26.9 | 11.3 | 10.8 | 52 | 32 | 47.3 | 72.6 | 0.02 |
| 9 | Ghotki | 24.5 | 22.2 | 21.4 | 51 | 19.6 | 43.1 | 55.4 | 0.09 |
| 10 | Sukkur | 20.9 | 27.2 | 26.9 | 66 | 27.9 | 72.8 | 69.4 | 0.14 |
| 11 | Khairpur | 22.4 | 19.7 | 18.7 | 56 | 29.8 | 55.5 | 90.8 | 0.17 |
| 12 | Naushahro Feroz | 21.7 | 20.7 | 18.5 | 67 | 62.5 | 53.4 | 75.8 | 0.01 |
| 13 | Shaheed Benazirabad | 22.8 | 23.9 | 22 | 57 | 36.4 | 63.7 | 76.8 | 0.04 |
| 14 | Dadu | 21.8 | 19.7 | 18.8 | 63 | 18.9 | 50.2 | 74.5 | 0.10 |
| 15 | Jamshoro | 20.7 | 21.9 | 21.4 | 57 | 68.5 | 57.2 | 75.3 | 0.06 |
| 16 | Hyderabad | 17.5 | 33.3 | 28.9 | 72 | 61.3 | 90.6 | 95 | 0.01 |
| 17 | Tando Allahyar | 21.4 | 28.8 | 26.1 | 53 | 63.8 | 67.4 | 91.7 | 0.09 |
| 18 | Tando Muhammad Khan | 21.2 | 28.5 | 26.4 | 38 | 32.1 | 71.8 | 79.9 | 0.08 |
| 19 | Sujawal | 26.2 | 15.9 | 15.3 | 33 | 40.2 | 52.6 | 79.3 | 0.06 |
| 20 | Thatta | 22.6 | 19.7 | 15.9 | 38 | 37.4 | 59.4 | 74.1 | 0.00 |
| 21 | Mirpurkhas | 23.9 | 24.5 | 21.9 | 43 | 38.1 | 51.8 | 61.9 | 0.02 |
| 22 | Umerkot | 22.4 | 19.2 | 19 | 32 | 60.1 | 37.3 | 55.8 | 0.04 |
| 23 | Tharparkar | 30.9 | 12.1 | 11.7 | 23 | 31.3 | 20.7 | 30.6 | 0.01 |
