# Supplementary material for: Effects of Integrating Family Planning With Maternal, Newborn, and Child Health Services on Uptake of Voluntary Modern Contraceptive Methods in Rural Pakistan: Protocol for a Quasi-experimental Study
Source: JMIR Res Protoc. 2022 Mar 8;11(3):e35291. doi: 10.2196/35291 (PMC8941439; doi:10.2196/35291)
Supplement: Multimedia Appendix 3 [file resprot_v11i3e35291_app3.docx]

**Multimedia Appendix 3: Operational Definitions**

| Primary Outcome Operational Definition [1] | |
| --- | --- |
| mCPR | Number of women age 15-49 years currently married who are using (or whose partner is using) a modern contraceptive method. (Modern methods include: male and female sterilization, injectable, intrauterine devices (IUDs), contraceptive pills, implants, male condoms, the standard days method, locational amenorrhea method, and emergency contraception) |
| Secondary Outcomes Operational Definition [1] | |
| Unmet need | Proportion of women who  (1) are not pregnant and not postpartum amenorrhea and are considered fecund and want to postpone their next birth for 2 or more years or stop childbearing altogether but are not using a contraceptive method, or  (2) have a mistimed or unwanted current pregnancy, or  (3) are postpartum amenorrhea and their last birth in the last 2 years was mistimed or unwanted. |
| Demand satisfied | Proportion of demand satisfied by modern methods:  Current contraceptive use (any modern method) divided by  Unmet need + current contraceptive use (any method) |
| Women attitude towards family planning | Proportion of women showing positive attitude towards FP |
| FP-MNCH integrated Services Coverage | Number of facilities providing FP-MNCH integrated Services |
| Utilization of FP-MNCH integrated services | Proportion of visits where clients received integrated FP- MNCH services (total number of Immunization client, total number of FP clients, total number of ANC and PNC clients) |
| Family planning referrals acceptors | Proportion of referral completers who accepted a FP method |
| Follow up for FP-MNCH services | Proportion of clients (women 15-49 and children U-5) visit for follow up who received integrated FP- MNCH services |
| Client satisfaction from FP-MNCH services | Percentage of women (15-49) satisfied with the services provided |
| Reasons for discontinuation /not using FP methods | Percentage distribution of reasons for not using FP methods among women (15-49) |
| Capacity Building | Percentage of trained staff:  Scoring 70% or above on knowledge post -assessment test using validated tools (facility and community level) |
| Unwanted pregnancy/births | Planning status of births/pregnancies Women reported whether their births/pregnancies were wanted at the time (planned birth), at a later time (mistimed birth), or not at all (unwanted birth).  Sample: Current pregnancies and births in the 5 years before the survey to women age 15-49 |
| Fertility rate and Age –specific fertility rate | The average number of children a woman would have by the end of her  Childbearing years if she bore children at the current age-specific fertility rates.  Age-specific fertility rates are calculated for the 3 years before the survey, based on detailed pregnancy histories provided by women. |
| Median birth interval | Percent distribution of non-first births by number of months since the preceding birth.  Categories correspond to birth intervals of <24 months, 24-35 months, 36-47  Months, and 48+ months. |
| Inter-pregnancy Interval | The number of months between a live birth and the conception of the next live birth. This was calculated by subtracting the “Date of last live birth” item from the date of birth to obtain a live-birth interval, then subtracting gestational age (months) of the birth from the live-birth interval. |
| Antenatal Coverage | Number of women age 15-49 years with a live birth in the last 2 years who were attended during their last pregnancy that led to a live birth  (1) at least once by skilled health personnel  (2) at least four times by any provider |
| Institutional Deliveries | Number of Institutional delivery by Skilled Birth attendant |
| PNC coverage Mother | Percentage of women age 15-49 who gave birth in the 2 years preceding the survey reported having a postnatal check in the first 2 days after the birth |
| PNC coverage Newborn | Percentage of mother age 15-49 who gave birth in the 2 years preceding the survey reported having a postnatal check for their newborn in the first 2 days after the birth |
| Immunization Coverage | Percentage of fully immunized children (12-23 months) |
| Increase Availability of Facility wise DHIS Data | Percentage of facility recording quality data to inform decision making at program and policy levels |

**References**

1. NIPS. National Institute of Population Studies (NIPS) [Pakistan] and ICF.Pakistan Demographic and Health Survey 2017-18. Islamabad`, Pakistan`, and Rockville`, Maryland`, USA: NIPS and ICF. 2017-2018. Available at <http://dhsprogram.com/pubs/pdf/FR354/FR354.pdf>.
