# Supplementary material for: Effects of Integrating Family Planning With Maternal, Newborn, and Child Health Services on Uptake of Voluntary Modern Contraceptive Methods in Rural Pakistan: Protocol for a Quasi-experimental Study
Source: JMIR Res Protoc. 2022 Mar 8;11(3):e35291. doi: 10.2196/35291 (PMC8941439; doi:10.2196/35291)
Supplement: Multimedia Appendix 4 [file resprot_v11i3e35291_app4.docx]

**Multimedia Appendix 4: Project Timeline**
